# Identification of the dopamine transporter SLC6A3 as a biomarker for patients with renal cell carcinoma

**DOI:** 10.1186/s12943-016-0495-5

**Published:** 2016-02-02

**Authors:** Sarah Schrödter, Martin Braun, Isabella Syring, Niklas Klümper, Mario Deng, Doris Schmidt, Sven Perner, Stefan C Müller, Jörg Ellinger

**Affiliations:** Department of Urology, University Hospital Bonn, Bonn, Germany; Section for Prostate Cancer Research, Institute of Pathology, Center for Integrated Oncology, University Hospital Bonn, Cologne/Bonn, Germany; Klinik und Poliklinik für Urologie und Kinderurologie, Universitätsklinikum Bonn, Sigmund-Freud-Strasse 25, 53105 Bonn, Germany

**Keywords:** Renal cell carcinoma, SLC6A3, Expression profiling, Biomarker, Microarray, Sertraline

## Abstract

**Background:**

Clear cell renal cell carcinoma (ccRCC) is among the most common human malignancies.

**Methods:**

In order to provide better understanding of the molecular biology of ccRCC and to identify potential diagnostic/prognostic biomarker and therapeutic targets, we utilized a microarray to profile mRNA expression of corresponding normal and malignant renal tissues. Real-time PCR, Western Blot and immunohistochemistry were applied to study the expression of candidate biomarkers. ccRCC cell lines were treated with sertraline to inhibit the dopamine transporter SLC6A3.

**Results:**

Differential expression of fourteen mRNAs, yet not studied in ccRCC in depth, was confirmed using qPCR (upregulation: SLC6A3, NPTX2, TNFAIP6, NDUFA4L2, ENPP3, FABP6, SPINK13; downregulation: FXYD4, SLC12A1, KNG1, NPHS2, SLC13A3, GCGR, PLG). Up-/downregulation was also confirmed for FXYD4, KNG1, NPTX2 and SLC12A1 by Western Blot on the protein level. In contrast to the mRNA expression, protein expression of the dopamine transporter SLC6A3 was lower in ccRCC compared to normal renal tissue. Immunohistochemistry indicated that this decrease was due to higher concentrations of SLC6A3 in the proximal tubules. Immunohistochemical analyses further demonstrated that high SLC6A3 expression in ccRCC tissue was correlated with a shorter period of recurrence-free survival following surgery. Treatment of ccRCC cells with the SLC6A3 inhibitor sertraline induced dose-dependent cell-death.

**Conclusion:**

Our study identified several novel biomarkers with diagnostic potential and further investigations on sertraline as therapeutic agent in ccRCC patients are warranted.

**Electronic supplementary material:**

The online version of this article (doi:10.1186/s12943-016-0495-5) contains supplementary material, which is available to authorized users.

## Background

Renal cell carcinoma (RCC) is one of the most common malignancies in developed countries: there are 115,200 new cases and 49,000 death estimated for 2012 in Europe [1]; and the incidence of RCC is increasing in the USA, especially in young patients and high-grade disease [2]. The widespread use of abdominal ultrasonography for check-up or clarification of non-specific symptoms led to an increased detection of small-sized renal tumors. Small renal tumors are non-malignant in up to one third, but imaging does often not allow precise identification of non-malignant tumors, and thus potential harmful overtherapy occurs often. While the resection of small RCC is usually curative, the prognosis of metastatic RCC is poor: surgery (cytoreductive nephrectomy, metastasectomy [3]) and targeted therapy [4] improved patients survival, but eventually most patients succumb to the disease.

From a clinical point of view, a non-invasive biomarker could help to reduce overtherapy in case of small renal tumors, and furthermore, could be helpful for clinical monitoring during medical tumor therapy. Despite extensive research during the past years, there is still no biomarker for RCC. RNA microarrays are a powerful tool to identify dysregulated genes in cancer tissue. We therefore applied a microarray to identify RNAs dysregulated in RCC patients. Genes showing differential expression were then validated using qPCR and Western Blot.

## Results

### Microarray: screening for aberrantly expressed mRNAs

**Fig. 1:**
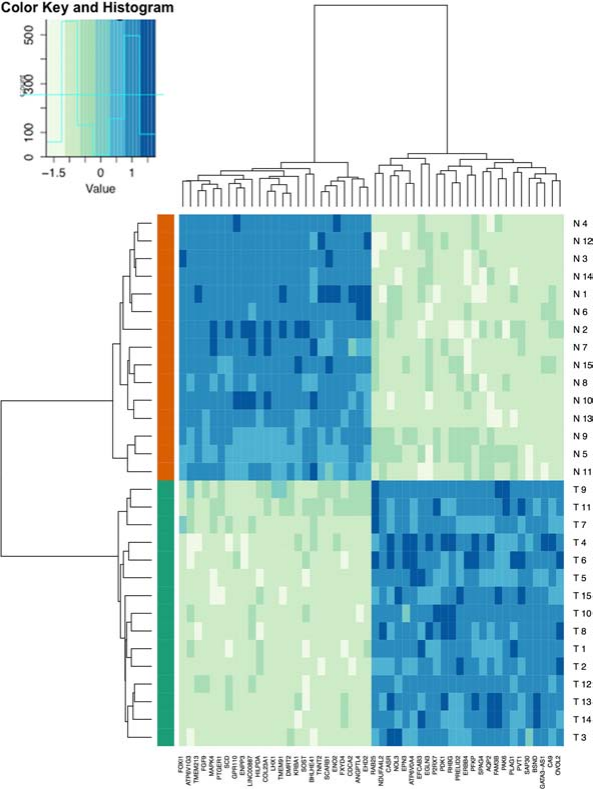
The expression of 34,144 mRNA transcripts was determined using a microarray in 15 corresponding tumor (red bar below heatmap) and normal (green) renal tissue samples. The mRNA expression profile allowed accurate discrimination of normal and clear cell renal carcinoma tissue; the heatmap demonstrates clustering of tissue samples according the expression of the 25 most up-/downregulated genes

The expression of 34,144 mRNA transcripts was determined using a microarray in 15 corresponding tumor and normal renal tissue samples. We observed differential expression, defined as log2-fold expression difference >2, in 1064 (3.1 %) transcripts: 368 mRNA transcripts were upregulated and 696 were downregulated in ccRCC samples. A hierarchical cluster analysis based on centered Pearson correlation coefficient was performed to determine different expression profiles in ccRCC and normal tissue. As shown in Fig. 1, the mRNA expression profile allowed to separate cancerous and normal tissue samples accurately. The most differentially expressed mRNAs in ccRCC and normal renal tissue are listed in Table 1. The Reactome database [5] was used to perform pathway analyses for these genes: affected pathways (FDR <0.05, *p* < 0.005) included glycolysis; glucose transport and metabolism; regulation of IGF transport; cellular response to hypoxia; regulation of HIF; transmembrane transport; lipid digestion, mobilization and transport. We further validated gene expression using 19 datasets comparing ccRCC and normal renal tissue from the NextBio tool [6]; as shown in Additional file 1 Figure S1, the dysregulation of these genes was also observed in former gene expression profiling studies.

**Table 1:** Summary of 25 most differentially expressed RNAs in clear cell renal cell carcinoma and normal tissue identified using microarray analysis

Downregulated in cancer			Upregulated in cancer		
Gene Name	Ensembl ID	Log2 fold-change	Gene Name	Ensembl ID	Log2 fold-change
AQP2	ENST00000199280	−10.63	CA9	ENST00000544074	7.34
UMOD	ENST00000199280	−9.94	NDUFA4L2	ENST00000555173	6.63
SLC12A1	ENST00000380993	−9.15	FABP7	ENST00000368444	6.09
KNG1	ENST00000432028	−8.69	LOC100131551	ENST00000414120	6.00
TMEM213	ENST00000442682	−8.56	NPTX2	ENST00000265634	5.98
CALB1	ENST00000469032	−8.54	ANGPTL4	ENST00000301455	5.92
ATP6V0A4	ENST00000310018	−8.13	CDCA2	ENST00000435898	5.61
FXYD4	ENST00000476166	−7.99	LOC100129597	uncharacterized	5.59
FABP1	ENST00000295834	−7.79	FABP6	ENST00000523955	5.49
ALDOB	ENST00000374855	−7.68	KISS1R	ENST00000234371	5.45
KLK1	ENST00000448701	−7.31	TNFAIP6	ENST00000460812	5.28
SLC13A3	ENST00000279027	−7.12	HILPDA	ENST00000466473	5.24
GCGR	ENST00000400723	−6.85	C10orf99	ENST00000472542	4.72
ATP6V1G3	ENST00000489986	−6.83	SLC6A3	ENST00000270349	4.59
MT1H	ENST00000332374	−6.75	COL23A1	ENST00000390654	4.59
RHCG	ENST00000536247	−6.70	SPINK13	ENST00000511106	4.58
NPHS2	ENST00000367615	−6.54	C3	ENST00000463520	4.47
PLG	ENST00000461414	−6.41	EGLN3	ENSG00000129521	4.44
SOST	ENST00000301691	−6.40	CYP2J2	ENST00000371204	4.40
FLJ45983	uncharacterized	−6.37	ENPP3	ENST00000358229	4.26
LHX1	ENST00000254457	−6.35	CD70	ENST00000245903	4.21
ALB	ENST00000430202	−6.35	NNMT	ENST00000299964	4.19
MT1G	ENST00000379811	−6.35	SLC17A4	ENST00000377905	4.15
AFM	ENST00000226355	−6.35	ANGPT2	ENST00000415216	4.10
SERPINA5	ENST00000537685	−6.32	HK2	ENST00000290573	4.08

### Real-Time PCR: validation of expression profiling

**Fig. 2:**
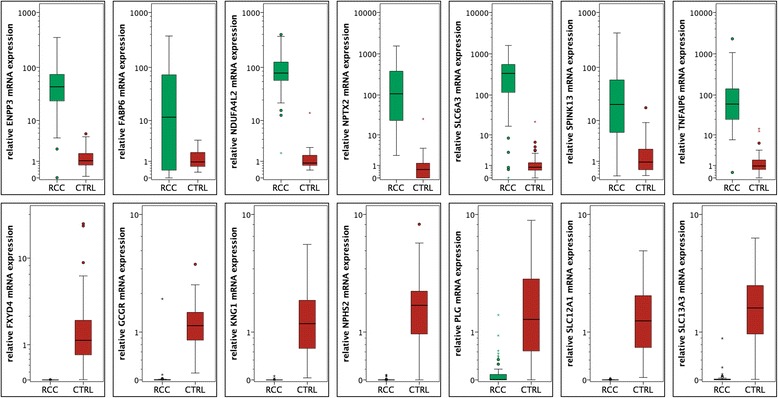
The expression of 14 target mRNAs in renal cell carcinoma (red bars) and normal renal (green bars) tissue was validated using quantitative real-time PCR; the expression levels were normalized using ACTB and TBP as reference genes. Significant overexpression of SLC6A3, NPTX2, TFAIP6, NDUFA4L2, ENPP3, FABP6 and SPINK13 mRNA in renal cell carcinoma was confirmed, whereas FXYD4, SLC12A1, KNG1, NPHS2, SLC13A3, GCGR and PLG mRNA levels were downregulated (all *p* < 0.001)

We next investigated the expression of 14 dysregulated mRNAs - not investigated by other researchers before - with an independent validation cohort of ccRCC (*n* = 52) and normal (*n* = 51) renal tissue samples using qPCR. As expected, we observed significant (all *p* < 0.001) upregulation of SLC6A3 (fold-change expression: mean 230-fold), NPTX2 (188-fold), TNFAIP6 (100-fold), NDUFA4L2 (78-fold), ENPP3 (42-fold), FABP6 (39-fold), and SPINK13 (26-fold) in ccRCC tissue. We also confirmed downregulation (all *p* < 0.001) of FXYD4 (mean 2371-fold), SLC12A1 (912-fold), KNG1 (758-fold), NPHS2 (407-fold), SLC13A3 (62fold), GCGR (27-fold), PLG (21-fold) in ccRCC tissue compared to normal renal tissue. Up- or downregulation allowed accurately identification of malignancy based on molecular signature: especially the loss of SLC12A1 was useful for diagnostic purposes as all malignant and benign samples were classified correctly (ROC analysis: sensitivity and specificity 100 %, area under the curve 1.0). See Table 2 and Fig. 2.

**Table 2:** Expression differences of selected mRNA in the validation cohort identified using qPCR

mRNA	fold change (mean)	cancer		normal		ROC analysis area under the curve
		mean	median	mean	median	
SLC6A3	230	379.006	335.639	1.645	0.810	0.955 (0.906-1.000)
NPTX2	188	246.544	107.117	1.314	0.595	0.993 (0.982-1.000)
TNFAIP6	100	170.493	60.204	1.701	0.944	0.980 (0.947-1.000)
NDUFA4L2	78	104.866	78.748	1.337	0.861	0.996 (0.989-1.000)
ENPP3	42	58.365	44.022	1.382	1.049	0.977 (0.939-1.000)
FABP6	39	49.888	11.631	1.279	0.948	0.677 (0.555-0.800)
SPINK13	26	51.221	20.516	1.948	0.939	0.894 (0.831-0.957)
FXYD4	−2371	0.001	0.000	2.371	1.179	0.998 (0.994-1.000)
SLC12A1	−912	0.002	0.000	1.596	1.352	1.000 (1.000-1.000)
KNG1	−758	0.002	0.000	1.602	1.259	1.000 (0.998-1.000)
NPHS2	−407	0.005	0.000	1.986	1.944	0.970 (0.936-1.000)
SLC13A3	−62	0.030	0.004	2.039	1.836	0.970 (0.943-0.997)
GCGR	−27	0.047	0.000	1.291	1.195	0.983 (0.951-1.000)
PLG	−21	0.113	0.006	2.406	1.401	0.927 (0.880-0.975)

We also determined whether mRNA expression levels in RCC patients were correlated with clinicopathological parameters. After correction for multiple hypothesis testing (Benjamini-Hochberg-procedure), we did not observe a significant association of any investigated mRNA and pT-stage, metastasis nor grading (all p > 0.03). However, there was a trend towards increased SLC6A3 levels in patients with metastases (p = 0.046). The mRNA levels were also not correlated with RCC recurrence nor survival, as determined using Cox regression analysis (all p > 0.1). We also retrieved the TCGA project data [7] via the cBio Cancer Genomics Portal [8]; SLC6A3 mRNA expression levels (RNA Seq V2 RSEM) were overexpressed in a subgroup of 5 % of the ccRCC samples and patients with high SLC6A3 expression in the tumor tissue had a shorter period of overall survival (median period of survival 40 vs. 75 months); See Additional file 1: Figure S2.

### Western Blot: validation on protein level

**Fig. 3:**
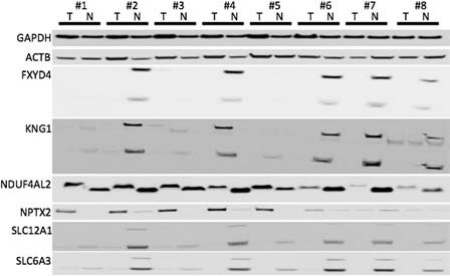
Western blot experiments were performed to determine the protein expression in eight corresponding normal (N) and clear cell renal cell carcinoma (T) tissue. NPTX2 was increased in tumor tissue, whereas FXYD4, KNG1, SLC12A1 and SLC6A3 were decreased in tumors. NDUF4AL2 levels were similar, but the bands were of different size in tumor and normal samples

The expression of FXYD4, SLC6A3, SLC12A1, NPTX2, NDUF4AL2 and KNG1 was determined in homogenized renal tissue (eight corresponding normal renal and ccRCC tissues) using Western Blot analyses. As shown in Fig. 3, NPTX2 expression was distinctly increased in tumor tissues. FXYD4, KNG1 (full length and the light chain) and SLC12A1 expression was decreased in ccRCC tissues. In contradiction to the mRNA expression, SLC6A3 and NDUF4AL2 levels were lower in tumor compared to normal renal tissues.

### Immunohistochemistry: expression of SLC6A3 in primary RCC

**Fig. 4:**
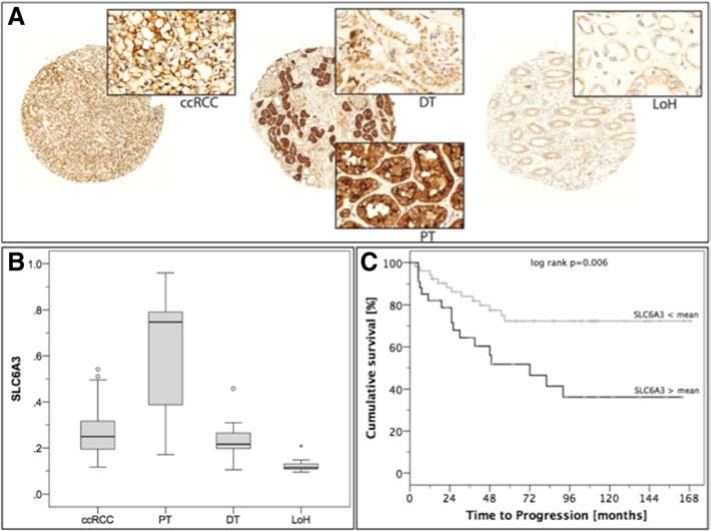
***a*** SLC6A3 expression was determined using immunohistochemistry in renal tissues; a representative image showing SLC6A3 immunohistochemical staining in ccRCC and normal renal tissue is shown. ***b*** The Boxplot diagram demonstrates the distribution of SLC6A3 expression in renal tissues: Nuclear, cytoplasmic and especially luminal-membranous SLC6A3 expression was high in proximal tubules (PT), whereas it was moderate in the distal tubules (DT) and clear cell renal cell carcinoma tissue (ccRCC); it was only weakly expressed in the loop of Henle (LoH). ***c*** SLC6A3 expression in tumor cells was predictive for progression-free survival: the period of progression-free survival was shorter in patients with SLC6A3 levels above the mean as shown in the Kaplan Meier estimates

In order to investigate the expression of SLC6A3 more detailed and in an enlarged cohort, we performed immunohistochemistry on a tissue microarray with 134 ccRCC and 21 normal renal tissues. SLC6A3 was strongly expressed at the membrane of tumor and normal cells, but also in the cytoplasm (see Fig. 4). In contrast to the mRNA expression, but in correspondence to the Western Blot analysis, SLC6A3 expression was significantly decreased in ccRCC tissue compared to normal renal tissue. SLC6A3 expression was low in the loop of Henle (both *p* < 0.0001), but high in the proximal tubules (*p* < 0.001) compared to ccRCC. Expression in ccRCC tissue and distal tubules was similar (p = 0.191). See Fig. 4.

SLC6A3 protein expression was increased in patients with distant metastasis (p = 0.002), but not correlated to AJCC stage, pT-stage, lymph node metastasis nor grading (all p > 0.1). Univariate Cox regression analysis indicated that an increased SLC6A3 immunostaining was correlated with poor progression-free survival (all p = 0.012; hazard ratio 6.7, 95 % confidence interval 3.4-18322.9); however, the predictive value was lost in a multivariate model which included pT-stage, lymph node/distant metastasis and grading (p = 0.365). The Kaplan-Meier estimate for the impact of SLC6A3 expression (stratified according the mean expression level in ccRCC) on progression-free survival is shown in Fig. 4.

### Determination of circulating SLC6A3 levels

The concentration of circulating SLC6A3 was determined using an ELISA assay. SLC6A3 was detected in serum of one patient with metastatic ccRCC (multiple bilateral lung metastases) at a concentration of 0.267 ng/ml. All remaining control subjects and ccRCC patients had SLC6A3 levels below the limit of detection (data not shown).

### Treatment of RCC cell lines with the SLC6A3 inhibitor sertraline

**Fig. 5:**
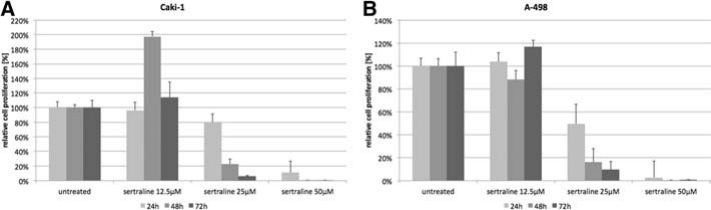
Caki-1 (**a**) and A-498 (**b**) cells were treated with the SLC6A3 inhibitor sertraline at 12.5 μM, 25 μM and 50 μM; cell proliferation decreased in a dose-dependent manner

We first determined SLC6A3 expression in ACHN, A498, Caki-1, Caki-2 and 769-P RCC cell lines; based on the SLC6A3 expression levels in the cell lines, we excluded ACHN, Caki-2 and 769-P from further analyses. Caki-1 and A498 cells were treated with the SLC6A3 inhibitor sertraline at different concentrations. Sertraline treatment resulted in a dose-dependent decrease of cellular proliferation with significant decrease at 25 μM and 50 μM doses. See Fig. 5.

## Discussion

In order to identify novel biomarkers for ccRCC, we investigated the mRNA expression profile in ccRCC using a microarray, and identified 1064 differentially expressed mRNA transcripts corresponding to 3 % of the investigated transcripts. As expected from earlier mRNA expression profiling studies in RCC patients, the expression profiling allowed a distinction of ccRCC and normal tissue accurately. The validity of the method was further confirmed by quantitative PCR experiments: 14 mRNA - so far not investigated by others - were quantified in a larger cohort (52 ccRCC and 51 normal renal tissues). Several other expression profiling [9–12] studies also observed deregulation of these genes. Finally, we confirmed the dysregulation of selected genes on the protein level.

The dopamine transporter SLC6A3 (solute carrier family 6 member 3, also termed DAT1) regulates dopamine concentrations in the brain and expression changes are associated with Parkinson’s syndrome and attention-deficit/hyperactivity disorder. However, the function of this transmembrane protein in the kidney and in carcinogenesis is unknown. We observed a distinct increase of SLC6A3 mRNA (>200-fold) in ccRCC tissue compared to the normal parenchyma indicating a role as tumor suppressor gene. Surprisingly, protein levels were increased in the normal renal tissue as measured using Western Blot. It should be noted that approximately 40 % of the genes have differential protein/mRNA levels, most probably due to various levels of regulation during protein synthesis, e.g. posttranscriptional, translational, or posttranslational regulation [13, 14]. Notably, genes involved in mRNA processing pathways (release of introns, 3’end processing, mRNA export) are altered in ccRCC [15]. The more detailed histological analysis demonstrated that especially the proximal tubules expressed SLC6A3 at high levels, whereas its levels were moderate in the tumor. Interestingly, SLC6A3 expression was correlated with the time to progression, as shown in the Kaplan Meier diagram. Notably, the multivariate Cox regression analysis failed to confirm the prognostic value, but the small size of the cohort (*n* = 88) limited the statistical power. Of interest, high SLC6A3 mRNA levels were predictive for shorter overall survival within the TCGA cohort [7]. Notably, Seyednasrollah et al. suggest that solute carrier genes are highly expressed in ccRCC and have an impact on ccRCC patients outcome [16]. We also treated RCC cell lines with sertraline, an inhibitor of the SLC6A3 protein, and observed a dose-dependent, significant decrease of cell proliferation. It was earlier shown that sertraline induces apoptosis in HepG2 hepatoma cells as a result of the activation of the TNF-MAP4K4-JNK cascade pathway [17]. Thus, sertraline - often used as antidepressant and pain drug in palliative care settings in cancer patients - could be a potential therapeutic agent in metastatic ccRCC. It should be noted that sertraline, despite of being the most potent antidepressant at the dopamine transporter, also inhibits the serotonin and norepinephrine transporter [18]. The SLC6A3 protein can be visualized in SPECT and PET using radiolabeled ligands [19], it may be speculated that SLC6A3 could be used to identify metastases from ccRCC.

The relevance of NPTX2 (neuronal pentraxin 2) expression in ccRCC was reported during later stages of our experiments: van Roemeling et al. [20] also observed upregulation of NPTX2 primary tumors and metastases. Re-evaluation of the TCGA dataset by Seyednasrollah et al. also revealed NPTX2 (among many other genes) as predictive for poor outcome, but an internal validation cohort failed to reproduce the prognostic role of NPTX2 [16]. It was shown that cell viability and cell migration were promoted by interaction with the AMPA receptor resulting in an influx of calcium into cancer cells. As NPTX2 expression was functionally characterized earlier, we did not perform knockdown experiments. Notably, we did not notice a correlation of NPTX2 expression and clinical-pathological parameters, whereas van Roemeling et al. suggested an increase of NPTX2 in more advanced ccRCC [20]. In pancreatic cancer, NPTX2 expression is silenced by DNA hypermethylation [21].

Finally, we observed downregulation of FXYD4m KNG1 and SLC12A1 in ccRCC tissue. FXYD4 (FXYD containing ion transport regulator 4) modulates the Na-K-ATPase [22]. FYXD4 has not been implicated in human carcinogenesis so far. SLC12A1 (solute carrier family 12 member 1) is a kidney specific sodium-potassium-chloride co-transporter; it plays a key role in sodium chloride resorption. SLC12A1 downregulation was also noticed by Liu et al. in ccRCC, and its expression levels were negatively correlated with miR-21 expression [23]. A pathogenic role in cancer was further reported in ovarian cancer cell lines, which exhibited increased SLC12A1 expression levels [24]. KNG1 (kininogen 1) plays an important role in blood coagulation, but is also observed in renal tissue where it acts proinflammatory via the production of eNOS, NO, cGMP and prostacyclin [25]. Blockage of KNG1 binding to endothelial cells inhibited angiogenesis in a colon cancer mouse model [26]. Increased KNG1 levels were detected in sera of patients with hepatocellular carcinoma [27].

It should be noted that the composition of our study cohorts was probably unsuited to identify genes with prognostic impact: the microarray experiment included only one patient with metastatic ccRCC; locally advanced RCC (pT3) was observed in 60 % of patients but most patients had low grade ccRCC (G3 13 %). Also, the PCR and tissue microarray validation cohorts included only 13 % patients with distant metastasis. Thus, the power to identify genes with prognostic impact may be limited.

## Conclusions

We have identified several novel biomarkers of potential diagnostic interest. The dopamine transporter SLC6A3 furthermore seems to be of prognostic interest, and could serve as therapeutic target in patients with metastatic ccRCC.

## Methods

### Patients

Fresh-frozen tissues from patients undergoing radical or partial nephrectomy were prospectively collected in the Biobank at the CIO Cologne Bonn at the University Hospital Bonn according to standard operating procedures. The renal tissue specimen were snap-frozen, and a cancerous and a normal sample from each patient was stored at -80 °C. To confirm the underlying histology, haematoxylin and eosin stained sections were made for review by an experienced uropathologist (S.P.). The 7^th^ edition of the TNM classification from 2009 was applied for RCC staging. We investigated a discovery cohort with 15 patients (ccRCC and adjacent normal renal tissue) to screen mRNA expression using microarray technology; an independent validation cohort with 52 ccRCC and 51 normal renal tissue samples was used for PCR experiments. The detailed clinical-pathological parameters are reported in Table 3. All patients gave written informed consent for the collection of biomaterials. The study was approved by the Ethikkommission at the Universitätsklinikum Bonn (number: 280/12).

**Table 3:** Clinical-pathological parameters of patients in the screening and validation cohort

	microarray screening cohort	PCR validation cohort		tissue microarray cohort
	*n* = 15 (%)	cancer *n* = 52 (%)	normal *n* = 51 (%)	*n* = 134 (%)
Sex				
male	10 (66.6)	35 (67.3)	35 (68.6)	85 (63.4)
female	5 (33.3)	17 (32.7)	16 (31.4)	49 (36.6)
Age				
mean	61.2	62.5	62.0	62.3
min-max	43-86	36-86	36-86	33-85
Pathological stage				
pT1	4 (26.7)	29 (55.8)	n.a.	55 (41.0)
pT2	2 (13.3)	5 (9.6)	n.a.	30 (22.4)
pT3	9 (60.0)	17 (32.7)	n.a.	47 (35.1)
pT4	0 (0)	1 (1.9)	n.a.	2 (1.5)
lymph node metastasis	0 (0)	2 (3.8)	n.a.	7 (5.2)
distant metastasis	1 (6.7)	7 (13.5)	n.a.	17 (12.7)
Fuhrman Grading				
grade 1	2 (13.3)	4 (7.7)	n.a.	40 (29.9)
grade 2	11 (73.3)	40(76.9)	n.a.	91 (67.9)
grade 3	2 (13.3)	7 (13.5 %)	n.a.	3 (2.2)
grade 4	0 (0)	1 (1.9)	n.a.	0

*n.a.* not applicable

### RNA isolation

RNA isolation of tissue samples was described earlier [28]: Approximately 50 mg tissue was homogenized, total RNA isolated using the mirVana miRNA Isolation Kit (Ambion, Foster City, CA, USA) and the RNA treated twice with DNase (DNA-free Kit, Ambion). The RNA quantity was measured (NanoDrop 2000 spectrophotometer; Thermo Scientific, Wilmington, DE, USA). RNA integrity was analyzed with a RNA 6000 n Kit on the Bioanalyzer 2100 (Agilent Technologies, Santa Clara, CA, USA); samples with a RIN > 6 were used for microarray experiments. RNA degradation was excluded by gel electrophoresis in samples used for PCR.

### Microarray analysis

The data from the microarray study were reported earlier (Gene Expression Omnibus database: GSE61763, [29]). In brief, microarray experiments were performed to determine the expression profile of 34,144 mRNA in 15 corresponding normal and ccRCC tissues. The microarray analysis was performed by Biogazelle (Zwijnaarde, Belgium) as a contract service. Background substracted and log2-based, normalized expression data were obtained for further data analysis. Based on normalized expression data, samples were gene wise scaled and centered, adjusting the inter-sample variance to 1. Differential expression was calculated pairwise, subtracting corresponding normal tissue from ccRCC tissue followed by studens *t*-test. For further analysis genes ranked with the highest (respectively lowest) differential expression value where taken into account, further p-value of the *t*-test for these genes had to be significant (*p* < 0.05). Clustering and heatmap aggregation, based on the top 25 up and 25 down ranked genes, was performed in euclidean space using complete linkage and gplot’s heatmap.2 function. Color coding was performed using the RColorBrewer Package. For all analyses R v3.2.0., with the packages mentioned has been performed.

### Real-Time PCR

To validate the microarray results, we investigated the expression of most differentially regulated mRNAs in an independent cohort (52 ccRCC and 51 normal renal tissue samples). cDNA was synthesized with 1 μg RNA using the PrimeScript RT Reagent Kit with gDNA Eraser. Real-Time PCR was performed with 5 ng cDNA template using the 1x SYBR Premix Ex Taq II with ROX Plus and 10 pmol/μl PCR primers; all reagents were from Takara Bio, Saint-Germain-en-Laye, France. The primer sequences are provided in Additional file 1: Table S1. PCR experiments were performed on an ABIPrism 7900 HT Fast Real-Time PCR System (Applied Biosystems, Foster City, CA, USA). Data were analyzed using Qbase + (Biogazelle) with ACTB and TBP, earlier shown to be suitable reference genes [30], in the 2-∆∆CT algorithm. Statistical analyses (Mann-Whitney-*U* test, Cox regression analysis) were performed with SPSS Statistics v21 (IBM, Ehningen, Germany).

### Western blot

Western Blot analyses were performed to determine the expression of six differentially expressed mRNAs on the protein level. We investigated eight corresponding ccRCC and normal renal tissues; the fresh-frozen tissue samples were located adjacent to the tissue used for RNA isolation. Approximately 50 mg tissue was homogenized in a Precellys 24 (Peqlab, Erlangen, Germany) with 400 μl Cell Lysis Buffer (Cell Signaling, Cambridge, United Kingdom) including protease inhibitor (Complete Mini EDTA-free, Roche, Basel, Switzerland). After determination of the protein concentration (BCA Protein Assay Kit, Pierce Biotechnology, Rockford, IL, USA), 30 ng protein was loaded per lane into a NuPAGE 3–8 % or 4–12 % denaturating PAA Gel (Life Technologies, Carlsbad, CA, USA) and separated in a XCell4 SureLock electrophoresis system (Life Technologies). A biotinylated protein ladder (catalog number 7727S, Cell Signaling) was used as molecular weight marker. After the transfer on 0.2 μm nitrocellulose (XCell II, Life Technologies) and a 5 % milk powder (Merck, Darmstadt, Germany) protein block, the immunostaining procedure was performed with antibodies against SLC6A3 (#LS-B7715, LSBio, Seattle, WA, USA), SLC12A1 (#NBP1-80993, Novus Bio, Cambridge, United Kingdom), FXYD4 (#NBP1-84489, Novus Bio), NPTX2 (#ab115528, Abcam, Cambridge, United Kingdom), KNG1 (#ab124737, Abcam), NDUF4AL2 (#ab190007, Abcam), beta-actin (#A5316, Sigma) and GAPDH (#2118, Cell Signaling). The detection was carried out with horseradish peroxidase conjugated to secondary antibodies (anti-mouse-POD, #170-6516, Biorad, Munich, Germany; anti-rabbit-POD, #170-6515, Biorad; anti-biotin-POD, #7075, Cell Signaling). The development of the chemiluminescent signal was done by SuperSignal West Femto (Pierce Technology) and documented by the LAS 3000 Image Reader (Fujifil, Tokyo, Japan).

### Immunohistochemistry

A tissue microarray [31] was used to determine the expression of SLC6A3 in ccRCC (*n* = 134) and normal renal tissue samples (*n* = 21). The expression pattern in the distal/proximal tubule and Henle’s loop was recorded separately. Paraffin sections of 5 μm thickness were cut from the tissue microarray block, and subsequently stained with an antibody against SLC6A3 (dilution 1:100). Immunohistochemical staining was performed using the Ventana Benchmark automated staining system (Ventana Medical System, Tuscon, AZ, USA). The slides were incubated with the primary antibody for 40 min at room temperature, whereby antibody dilution was conducted with the Ventana diluent. Signal detection was performed with the Ventana DABMap Kit combined with Universal Secondary Antibody. The slides were finally counterstained with haematoxylin and Bluing Reagent, dehydrated and mounted.

Each slide was scanned by the Panoramic Midi (3D HISTECH, Budapest, Hungary). An image analysis software (Tissue Studie v2.1; Definiens, Munich, Germany) was used to obtain a continuous spectrum of average staining intensity as reported before [32]. The staining intensities were statistically evaluated using SPSS Statistics v21; the SPSS Mann-Whitney-*U* test was applied to compare SLC6A3 expression in normal and tumor tissues. Kaplan Meier estimates and Cox regression analyses were performed to determine the relevance of SLC6A3 expression for progression-free, cancer specific and overall survival.

### SLC6A3 ELISA

We used a commercial enzyme linked immunosorbent assay (ELISA) kit for SLC6A3 (Human Dopamine Transporter ELISA Kit, #DL-DAT-Hu; Wuxi Donglin Sci & Tech Development, Jiangsu, China) to determine the concentration of circulating SLC6A3 in ccRCC patients. The ELISA was performed with 100 μl serum essentially as recommended by the supplier. The optical density was recorded with a Safire photometer (Tecan, Männedorf, Switzerland) at 450 nm. Data analysis was performed with the Magellan Software v7.2. We examined 15 healthy individuals and 18 patients with ccRCC (stage I: *n* = 10; stage II: *n* = 3; stage III: *n* = 3, stage 4: *n* = 2). All samples were measured in duplicate.

### Cell culture and sertraline treatment

The RCC cell lines Caki-1 and A498 were purchased from DSMZ (Braunschweig, Germany). Cells were grown in RPMI 1640 supplemented with 10 % heat-inactivated fetal calf serum, glutamine (PAA, Pasching, Austria) and 1 % penicillin/streptomycin (Invitrogen, Paisley, Scotland) and incubated at 37 °C, 5 % CO2. The cell lines were treated with 0 μM, 12.5 μM, 25 μM and 50 μM sertraline (SLC6A3 inhibitor) for up to 72 h in six replicates in 96-well tissue culture plates, which contained 1.5x10^4^ cells/well. Cells were cultured up to 72 h to assess cell viability using the EZ4U test.

## Additional file

Additional file 1:
**List of qPCR primer sequences used for the validation study.** (PDF 811 kb)
