# Supplementary material for: Identification of the dopamine transporter SLC6A3 as a biomarker for patients with renal cell carcinoma
Source: Mol Cancer. 2016 Feb 2;15:10. doi: 10.1186/s12943-016-0495-5 (PMC4736613; doi:10.1186/s12943-016-0495-5)

Identification of the dopamine transporter SLC6A3 as biomarkers for patients with renal cell carcinoma

Sarah Schrödter; Martin Braun; Niklas Kümper; Mario Deng; Isabella Syring; Doris Schmidt; Sven Perner; Stefan C Müller; Jörg Ellinger

Supplementary Table S1

List of qPCR primer sequences used for the validation study.

Gene Symbol	Ensembl ID	Forward Primer	Reverse Primer
NDUFA4L2	ENST00000555173	CGCTTTACTTGCTGCGACT	GGCTCCGGGTTGTTCTTT
NPTX2	ENST00000265634	GAGAAGTCCCTGCTGCACAA	CTATTGCCTCGCTCCAGCTC
FABP6	ENST00000523955	TGAGAGCTGTGTTGTCTGCG	CTGCTGGGAGGCTTTCCTTT
TNFAIP6	ENST00000460812	GGCCCAACTGTGGATTTGGA	TCCTTTGCGTGTGGGTTGTA
SLC6A3	ENST00000270349	TCACCAACGGTGGCATCTAC	CACTCCGATGGCTTCGATGA
SPINK13	ENST00000511106	CCACCTCATCCAGGCCTTATC	AATCTTGTGGGGAAAGGCAG
ENPP3	ENST00000358229	TGTCACGGGCTTGTATCCAG	TGCCACCAGGCTGGATTATT
SLC12A1	ENST00000380993	CCATGCCTCTTATGCCAAAT	CACATGTTGTAAATTCCATACGC
KNG1	ENST00000432028	TCAACCACTGGGAATGATCTCAC	TCGCAGGACCTTAGGTGACTA
FXYD4	ENST00000476166	CTCGCAGGACACTGGTGAAG	TGCTTGTGGGGAGATCTTGG
SLC13A3	ENST00000279027	TTGCTGGTACAAAGACATGGT	TGGTCTGTGCCCAGGTATTC
GCGR	ENST00000400723	CCGCGCGGACCCTGA	GAGTGGCAGAGCAGCAGAG
NPHS2	ENST00000367615	GCCCGAGGAAGGTACCAAAT	CGCAGAACCAGATGGAAAAAGG
PLG	ENST00000461414	AACAAGCGCTGGGAACTTT	CACTGGTAGGTGGGACCAGA
PPIA	ENST00000321231	ATGCTGGACCCAACACAAAT	TCTTCACTTTGCCAAACACC
ACTB	ENST00000462494	CCACCGCGAGAAGATGA	CCAGAGGCGTACAGGGATAG
TBP	ENST00000230354	GAACATCATGGATCAGAACAACA	ATAGGGATTCCGGGAGTCAT

Supplementary Figure S1

Visualization of the gene expression data retrieved from the NextBio database.

NDUF4AL2	TNFAIP6	NPTX2	ENPP3	SLC6A3	FABP6	SPINK13	GCGR	PLG	SLC13A3	FXYD4	NPHS2	SLC12A1	KNG1	NextBio Bioset ID	Accession
5,2	5,3	5,2	2,9	3,0	3,4	2,5	n.s.	-4,1	-4,8	-5,5	-6,8	-7,2	-7,7	702791	GSE36895
5,0	5,6	5,6	3,1	3,7	2,6	1,7	-0,6	-4,7	-4,9	-4,4	-5,8	-7,4	-7,5	830047	GSE53757
5,1	3,2	4,7	2,5	1,8	2,3	2,1	-1,0	-3,5	-2,6	-4,5	-3,5	-7,5	-6,4	68893	GSE14762
5,8	5,0	5,4	4,8	3,5	1,8	n.s.	-1,1	n.s.	-2,2	-4,4	-3,0	-6,6	-6,2	451960	GSE26574
5,9	5,3	5,1	4,7	3,5	1,7	n.s.	-0,8	-2,4	-3,2	-4,2	-3,5	-6,6	-6,5	153337	GSE11024
5,1	n.s.	5,3	4,6	2,6	3,1	3,6	-0,7	n.s.	n.s.	-4,9	n.s.	-9,0	-8,2	53136	GSE12606
4,7	3,9	3,6	3,1	2,8	1,1	0,6	-0,6	-2,8	-2,9	-3,8	-3,2	-6,0	-5,4	829438	GSE46699
5,3	3,4	4,3	3,0	3,2	2,5	1,4	-4,0	-4,7	-4,0	-4,8	-4,2	-1,9	-6,3	772343	GSE40435
6,4	5,3	5,2	n.s.	2,1	3,4	n.s.	-0,6	-4,9	-5,3	n.s.	-6,3	-6,0	-7,5	84736	GSE14994
6,2	5,0	3,0	n.s.	2,3	2,7	n.s.	-0,7	-4,3	-5,6	n.s.	-6,5	-6,4	-8,1	84724	GSE14994
3,6	3,8	2,6	n.s.	1,8	2,6	n.s.	n.s.	-4,9	-4,8	n.s.	-6,5	-6,6	-7,2	84730	GSE14994
3,6	6,8	5,2	3,2	3,4	2,1	1,0	n.s.	-6,5	-5,3	-3,6	-7,0	-6,3	-8,0	52172	E-TABM-282
4,6	3,1	3,5	n.s.	2,0	2,1	n.s.	-0,5	-2,2	-2,7	n.s.	-3,4	-5,7	-6,0	79486	GSE15641
6,3	4,7	4,4	2,3	3,4	n.s.	n.s.	n.s.	-6,0	-6,7	-3,8	-6,0	-6,2	-7,1	37448	GSE781
n.s.	7,4	5,2	5,0	n.s.	n.s.	1,7	n.s.	-1,4	-5,8	-7,5	n.s.	-10,0	-8,0	56278	GSE4125
5,5	5,4	3,2	3,7	2,4	0,5	n.s.	-0,7	-4,0	-3,5	-3,1	-3,9	-4,1	-5,0	364384	GSE6344
5,2	3,9	n.s.	2,8	n.s.	2,1	n.s.	-0,6	-3,7	-4,0	-3,2	-3,8	-4,2	-5,7	364381	GSE6344
5,5	6,0	5,1	4,6	6,1	5,9	4,0	-2,3	-3,2	-4,8	-6,2	-7,1	-8,4	-5,6	853948	TCGA
2,7	4,4	1,5	3,9	2,8	1,4	n.s.	-0,9	-3,4	-3,8	-3,7	-4,7	-6,9	-6,4	821530	GSE53000

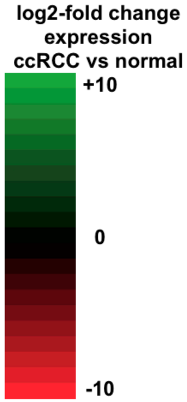

Supplementary Figure S2

Kaplan Meier estimate for SLC6A3 expression and overall survival in patients with clear cell renal cell carcinoma from The Cancer Genome Atlas (TCGA) cohort using the cBio Cancer Genomics Portal. Patients with higher expression levels of SLC6A3 (n=26; 5%) have a poor outcome (p=0.005). Note that overexpression was defined as higher expression levels within the tumor tissues and not compared to normal tissue in the cBioPortal.

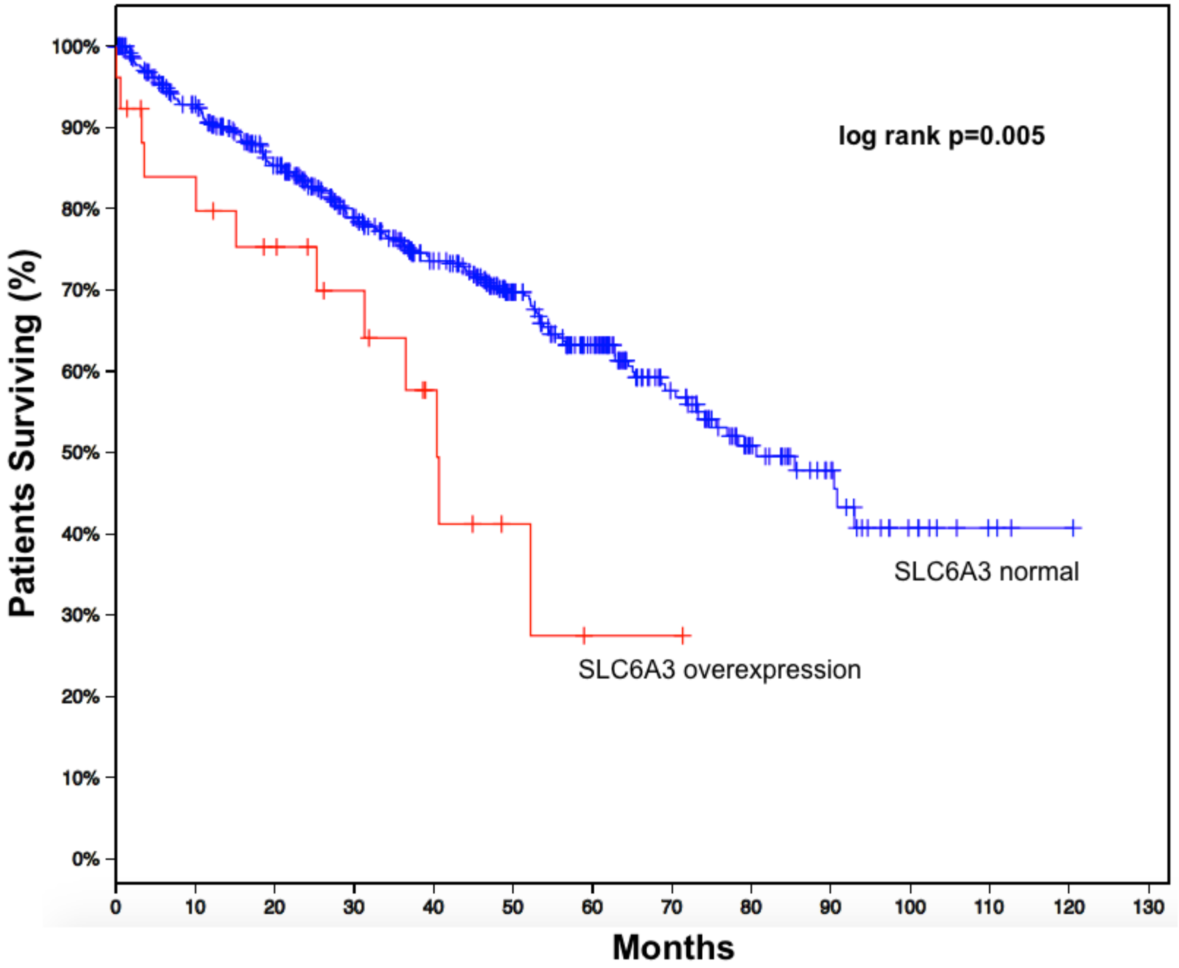

Supplement: Additional file 1: — List of qPCR primer sequences used for the validation study. (PDF 811 kb) [file 12943_2016_495_MOESM1_ESM.pdf]
